# Direct and Indirect Effectiveness of mRNA Vaccination against Severe Acute Respiratory Syndrome Coronavirus 2 in Long-Term Care Facilities, Spain

**DOI:** 10.3201/eid2710.211184

**Published:** 2021-10

**Authors:** Susana Monge, Carmen Olmedo, Belén Alejos, María Fé Lapeña, María José Sierra, Aurora Limia, COVID-19 Registries Study Group

**Affiliations:** Ministry of Health, Madrid, Spain

**Keywords:** COVID-19, coronavirus disease, SARS-CoV-2, severe acute respiratory syndrome coronavirus 2, viruses, respiratory infections, zoonoses, vaccination, vaccine effectiveness, long-term care facilities, elderly, indirect effects, transmissibility, Spain

## Abstract

We conducted a registries-based cohort study of long-term care facility residents >65 years of age offered vaccination against severe acute respiratory syndrome coronavirus 2 before March 10, 2021, in Spain. Risk for infection in vaccinated and nonvaccinated persons was compared with risk in the same persons in a period before the vaccination campaign, adjusted by daily-varying incidence and reproduction number. We selected 299,209 persons; 99.0% had >1 dose, 92.6% had 2 doses, and 99.8% of vaccines were Pfizer/BioNTech (BNT162b2). For vaccinated persons with no previous infection, vaccine effectiveness was 81.8% (95% CI 81.0%–82.7%), and 11.6 (95% CI 11.3–11.9) cases were prevented per 10,000 vaccinated/day. In those with previous infection, effectiveness was 56.8% (95% CI 47.1%–67.7%). In nonvaccinated residents with no previous infection, risk decreased by up to 81.4% (95% CI 73.3%–90.3%). Our results confirm vaccine effectiveness in this population and suggest indirect protection in nonvaccinated persons.

From the beginning of the coronavirus disease (COVID-19) pandemic through March 7, 2021, a total of 18,927 residents in long-term care facilities (LTCF) in Spain died from confirmed COVID-19, resulting in a cumulative mortality rate of 67/1,000 residents. An additional 10,492 persons have died exhibiting symptoms compatible with COVID-19 (*1*). Dependent persons living in closed institutions are at higher risk for exposure. In addition, older age and underlying conditions are associated with more severe infection. Indeed, in the LTCF setting, death was the outcome of 1/5 cases of severe acute respiratory syndrome coronavirus 2 (SARS-CoV-2) infection (*1*).

In Spain, COVID-19 vaccination with the Pfizer/BioNTech (BNT162b2; https://www.pfizer.com) vaccine began on December 27, 2020; LTCF residents and workers were the first priority group (*2*). The vaccination campaign coincided with the third COVID-19 epidemic wave in Spain; national 14-day cumulative incidence increased from <250 cases/100,000 population at the end of 2020 to >1,000 by the end of January 2021 (*3*). Vaccination started in facilities considered at higher risk, such as those that had never experienced a COVID-19 outbreak, had higher numbers of residents, or had experienced more difficulties implementing prevention and control measures. Vaccination teams visited the facilities and vaccination was offered to all, including persons with previous SARS-CoV-2 infection. Vaccination was deferred only in persons with active infection. The recommendation was to vaccinate persons under quarantine, but this guidance was inconsistently followed by vaccination teams. Acceptance has been high; 97.8% of all LTCF residents received >1 vaccine dose, and 88.8% received 2 doses (*4*).

The Pfizer/BioNTech vaccine has shown an efficacy of 95% in preventing COVID-19 in randomized clinical trials (*5*). However, elderly persons in general, and those living in LTCF in particular, are not well represented in randomized studies (*6*). Therefore, interest in estimating vaccine effectiveness (VE) in this population after widespread vaccination is great. Moreover, because vaccination coverage was so high, nonvaccinated persons might be indirectly protected if vaccination reduces infection and transmissibility among vaccinated persons. A few observational studies focusing on the elderly have been published recently; 2 published and 1 preprint studies have specifically addressed effects of vaccination in LTCF residents (*7,8*; I.R. Moustsen-Helms et al., unpub. data, https://www.medrxiv.org/content/10.1101/2021.03.08.21252200v1). However, none have addressed indirect protection in nonvaccinated persons in this high-coverage setting. This study aims to estimate indirect and total (direct plus indirect) effects of vaccination in residents of LTCFs in Spain in a high-incidence context.

## Methods

### Data Sources

REGVACU (Registro de Vacunación COVID-19) is a nationwide registry of all COVID-19 vaccine doses administered and vaccine rejections in Spain. Administrative censoring was on March 10, 2021. We selected persons who were >65 years of age by December 27, 2020, with a valid postal code who were identified as residents in elderly homes according to REGVACU. SERLAB (Sistema  Estatal  de  Resultados  de  Laboratorio) is a nationwide registry of all SARS-CoV-2 PCR and rapid antigen tests performed. We excluded positive tests within 60 days of a previous positive result, because they were more likely to correspond to prolonged PCR positivity than to reinfection, according to national guidelines (*9*). In LTCFs, tests were performed on symptomatic persons and at-risk contacts. Incoming residents were also routinely tested and periodic screenings were conducted. Therefore, documented infections registered in SERLAB reflect symptomatic and asymptomatic infections, although this distinction was not recorded. Residents in REGVACU were cross-matched with SERLAB by person identification number, birthdate, and sex.

### Study Design

**Figure 1:**
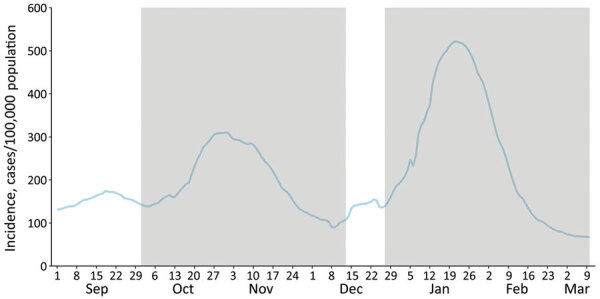
Seven-day cumulative incidence of diagnosis of severe acute respiratory syndrome coronavirus 2, Spain. Shadowed areas indicate the study period for the selected persons (December 27, 2020–March 10, 2021) and the reference period 87 days before (October 1–December 13, 2020).

To estimate the effect of vaccination in vaccinated persons, we studied the risk for documented SARS-CoV-2 infection in the cohort of persons in whom the first dose was administered during December 27, 2020–March 10, 2021 (study period). We considered a before-after comparison more appropriate because of the possibility of indirect protection in nonvaccinated persons after the vaccination program began, because of the high vaccination coverage achieved at LTCFs. This effect would mean that nonvaccinated persons (after the start of the vaccination program) would not represent infection risk in the absence of vaccination. Therefore, for the comparison group, we included the same persons but in the period before the vaccination program started. Baseline infection risk, on the other hand, is heavily influenced by community incidence, and the study period coincided with the third epidemic wave in Spain. To minimize this effect, we chose as reference period the second epidemic wave. In particular, the most comparable period in terms of COVID-19 incidence was October 1–December 13; start dates for the reference period and study period were 87 days apart (Figure 1).

To estimate the indirect protection of vaccination in unvaccinated persons, we compared the risk for documented SARS-CoV-2 infection in the cohort of nonvaccinated persons in the study period with risk in the same persons during the reference period, similarly to the method explained previously for vaccinated persons. Because all residents of the same LTCF were offered vaccination on the same day, the follow-up period began on the date when the vaccine was first offered. Therefore, we could ensure that persons were included on the date that a first vaccine dose was administered to most coresidents and workers.

We also registered any previous documented SARS-CoV-2 infection on the first day of follow-up in the reference period or the study period for both analyses. Follow-up for all persons in the study period or reference period concluded if the person tested positive for SARS-CoV-2 or at administrative censoring (December 13 for the reference period or March 10 for the study period), whichever occurred first. Unfortunately, no information on deaths was available.

To monitor for possible design-associated bias, we created a bias-indicator cohort with nonvaccinated time-at-risk during the study period of persons who were later vaccinated, with follow-up beginning on December 27 and ending at date of first vaccine dose or date of positive SARS-CoV-2 test. We compared it to equivalent follow-up time in the reference period, similarly to the method explained previously. The study was approved by the research ethics committee at the Instituto de Salud Carlos III (approval no. CEI PI 98_2020).

### Data Analysis

We performed analyses separately for the group with previously documented SARS-CoV-2 infection and the group with no previously documented infection. We computed the standardized cumulative risk for a documented SARS-CoV-2 infection under a causal inference approach (*10*). First, to estimate the probability of the event on each follow-up day, conditioned to remaining event-free up to that day and given the individual covariates, a pooled logistic regression was fitted adjusting by follow-up day, daily varying 7-day SARS-CoV-2 cumulative incidence specific to the province, its quadratic term, and the empirical reproduction number for that province on that date. An interaction between follow-up day and vaccination was introduced to allow for a time-varying effect of the vaccine. We built robust models by using individual identification as a clustering variable. We predicted the probability of the event by using this model for 2 contrafactual samples: one in which everyone was exposed, as defined for the study period (vaccinated persons or, for the indirect effect analysis, nonvaccinated persons indirectly protected) and one in which everyone was unexposed, as in the reference period. We then used the Kaplan-Meier method to derive standardized cumulative risk curves.

Risk ratios (RR) comparing the risk in the exposed and the unexposed, VE (VE = 1 – RR), and risk difference (RD) were estimated for the overall period and in 4 subperiods after the administration of the first dose to serve as proxies of different vaccine protection: 0–14 days, 15–21 days, 22–28 days (proxy of first 7 days after the second dose), and >29 days (proxy of fully vaccinated [i.e., >7 days after second vaccine dose]). We estimated normal distribution-based 95% CIs by using bootstrapping with 300 repetitions.

## Results

### Description of Participants

Out of 5,068,733 vaccination records from 3,615,403 persons in REGVACU before March 10, a total of 573,533 records from 299,209 persons were selected as being >65 years of age, having a valid postal code, and living in a LTCF. Of those, 296,093 (99.0%) had received >1 vaccine dose, of which 99.8% were Pfizer/BioNTech (BNT162b2) and 0.2% were Moderna (https://www.modernatx.com); 92.6% received a second vaccine dose within a median of 21 days (interquartile range [IQR] 21–21 days); 3,116 (1.0%) were not vaccinated (Appendix Figure 1). Mean (SD) age was 85.9 (+7.8) years and 70.9% were women. We cross-matched selected persons with SERLAB; 77,662 (26.0%) had >1 positive test during March 1, 2020–March 11, 2021.

A previous SARS-CoV-2 infection was identified in 12.7% of vaccinated participants at the beginning of the reference period and 22.3% at the beginning of the study period. The median time since the last positive SARS-CoV-2 test (PCR or rapid antigen test) was 173 (IQR 48–189) days at the beginning of the reference period and 106 (IQR 57–264) days in the study period. Similarly, in the indirect effects analysis, 27.7% of nonvaccinated persons had previous infection at the beginning of follow-up in the study period and 12.9% had previous infection in the reference period. The median time since the last positive SARS-CoV-2 test in this group was 179 (IQR 62–191) days at the beginning of the reference period and 76 (IQR 44–264) days in the study period.

### Estimation of VE in Persons with No Previous SARS-CoV-2 Infection

**Figure 2:**
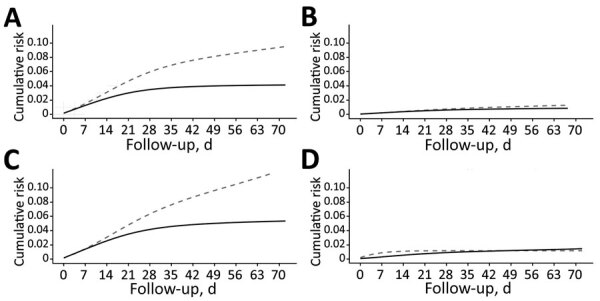
Cumulative incidence of documented severe acute respiratory syndrome coronavirus 2 infection in long-term care facilities estimated from adjusted hazards models, Spain, December 27, 2020–March 10, 2021. A) Standardized risk in the vaccinated with no previous infection and its reference group; B) standardized risk in the vaccinated with previous infection and its reference group; C) standardized risk in nonvaccinated (indirectly protected) with no previous infection and its reference group; D) standardized risk in nonvaccinated (indirectly protected) with previous infection and its reference group. Solid lines indicate study group; dotted lines indicate reference group.

VE in vaccinated persons without evidence of previous SARS-CoV-2 infection was estimated on the basis of 230,195 persons vaccinated during the study period and 258,357 persons in the reference period. A total of 10,785 events occurred during the study period, and 19,244 events occurred during the reference period (Table 1; Appendix Table 1, Figure 2). Adjusted VE for the study period was 57.6% (95% CI 56.6%–58.6%), which increased after full vaccination to 81.8% (95% CI 81.0%–82.7%) (Table 1; Figure 2). The estimated number of SARS-CoV-2 infections averted by vaccination (RD) was greatest in the intermediate periods, which coincided with the peak of the epidemic waves at 11.6 cases/10,000 vaccinated persons per day.

**Table 1:** Standardized risk, risk ratio, vaccine effectiveness, and risk difference in elderly residents of long-term care facilities with no evidence of previous severe acute respiratory syndrome coronavirus 2 infection, by time since first vaccinated, Spain, December 27, 2020–March 10, 2021*

Time since vaccination	Events/persons at risk			Standardized risk† (95% CI)		RR (95% CI)	VE, % (95% CI)	RD (95% CI)
Reference period	Study period	Unexposed	Exposed
Effects in the vaccinated								
Full period	19,244/258,357	10,785/230,195		12.8 (12.61–12.98)	5.42 (5.32–5.52)	0.43 (0.41–0.42)	57.6 (56.6–58.6)	−7.37 (−7.58 to −7.16)
0–14 d	5,355/258,357	5,957/230,195		20.92 (20.49–21.33)	14.87 (14.56–15.16)	0.73 (0.69–0.71)	28.9 (26.9–31)	−6.05 (−6.56 to −5.54)
15–21 d	2,966/246,924	2,690/218,621		22.34 (21.97–22.72)	10.75 (10.55–10.95)	0.49 (0.47–0.48)	51.9 (50.7–53.1)	−11.59 (−12.01 to −11.19)
22–28 d	3,234/239,409	1,253/212,421		18.43 (18.14–18.72)	6.84 (6.67–7.0)	0.38 (0.36–0.37)	62.9 (61.9–64)	−11.59 (−11.92 to −11.28)
>29 d‡	7,389/230,438	885/207,774		7.91 (7.73–8.09)	1.44 (1.37–1.49)	0.19 (0.17–0.18)	81.8 (81.0–82.7)	−6.47 (−6.66 to −6.28)
Indirect effects in the unvaccinated								
Full period	271/2,713	117/2,254		17.16 (15.07–19.21)	7.08 (5.79–8.35)	0.41 (0.32–0.51)	58.7 (49.4–68.5)	−10.08 (−12.62 to −7.52)
0–14 d	70/2,713	59/2,254		20.87 (17.54–24.02)	17.08 (13.68–20.48)	0.82 (0.6–1.03)	18.2 (−3.1 to 39.8)	−3.79 (−8.54 to 1.14)
15–21 d	37/2,565	22/2,128		24.51 (21.37–27.52)	13.48 (11.11–15.91)	0.55 (0.43–0.67)	45 (32.8–57.1)	−11.02 (−14.88 to −6.99)
22–28 d	38/2,473	16/2,056		22.16 (19.34–24.93)	9.35 (7.36–11.37)	0.42 (0.32–0.53)	57.8 (47.5–68.2)	−12.81 (−16.16 to −9.39)
>29 d‡	126/2,350	20/1,997		14.09 (11.46–16.73)	2.63 (1.58–3.62)	0.19 (0.1–0.27)	81.4 (73.3–90.3)	−11.46 (−14.39 to −8.6)

*Time since first vaccinated was a proxy of number of vaccine doses and days since last dose. RD, risk difference; RR, risk ratio; VE, vaccine effectiveness. †Per 10,000 population per day. ‡Full vaccination.

We estimated indirect effects of vaccination in nonvaccinated persons without evidence of previous SARS-CoV-2 infection on the basis of 2,713 persons not vaccinated during the study period and 2,254 nonvaccinated persons in the reference period. Within these groups, 271 events occurred during the study period and 117 events occurred during the reference period (Appendix Table 1, Figure 2). Adjusted indirect protection was estimated at 58.7% (95% CI 49.4%–68.5%) for the whole study period. There was no statistically significant reduction in risk in the first 14 days of follow-up, but protection increased progressively thereafter, particularly after >29 days (as a proxy of full vaccination in LTCF residents), when VE reached 81.4% (95% CI 73.3%–90.3%) (Table 1; Figure 2). The estimated number of SARS-CoV-2 infections averted by vaccination was similar to that found in the vaccinated group.

### Estimation of VE in Persons with Previous SARS-CoV-2 Infection

VE in vaccinated persons with previous SARS-CoV-2 infection was estimated on the basis of 65,898 persons vaccinated during the study period, and 37,736 persons in the reference period. A total of 519 events occurred during the study period, and 412 events occurred during the reference period (Table 2). Time since previous infection to the beginning of follow-up was similar for those in whom an event occurred (median 129 [IQR 72–187] days) or those who remained event-free (median 134 [IQR 55–208] days) (Appendix). Baseline risk in those with previous infection was 1.78 (95% CI 1.58–1.96) infections/10,000 persons/day, much lower than the baseline risk in those with no previous infection of 12.8 (95% CI 12.6–13.0) infections/10,000 persons/day (Tables 1, 2). Consequently, VE was lower than that seen for those with no history of previous infection (Appendix Table 1, Figure 2). Adjusted VE for the whole study period was 36.3% (95% CI 27.9%–45.5%), which increased after full vaccination to 56.8% (95% CI 47.1%–67.7%) (Table 2; Figure 2); the number of infections averted was lower at ≈0.7/10,000 vaccinated persons/day. Estimating VE for indirect protection in the group with previous SARS-CoV-2 infection was not possible because only 14 events occurred (Table 2), 95% CIs virtually tended to infinite, and the model did not result in credible risk curves.

**Table 2:** Standardized risk, risk ratio, vaccine effectiveness, and risk difference in residents of long-term care facilities with evidence of previous severe acute respiratory syndrome coronavirus 2 infection, by time since first vaccinated, Spain, December 27, 2020–March 10, 2021*

Time since vaccination	Events/persons at risk			Standardized risk† (95% CI)		RR (95% CI)	VE (95% CI)	RD (95% CI)
Reference period	Study period	Unexposed	Exposed
Effects in the vaccinated								
Full period	412/37,736	519/65,898		1.78 (1.58–1.96)	1.13 (1.02–1.23)	0.64 (0.54–0.72)	36.3 (27.9–45.5)	−0.64 (−0.86 to −0.44)
0–14 d	100/37,736	245/65,898		2.64 (2.22–3.03)	2.39 (2.13–2.63)	0.9 (0.73–1.07)	9.6 (−6.9 to 26.8)	−0.25 (−0.72–0.23)
15–21 d	72/37,440	104/64,988		2.58 (2.26–2.89)	1.92 (1.74–2.09)	0.74 (0.63–0.85)	25.5 (15.1–36.6)	−0.66 (−1.00 to −0.32)
22–28 d	77/36,840	55/63,236		2.2 (1.95–2.44)	1.44 (1.29–1.58)	0.65 (0.56–0.74)	34.6 (25.7–44.1)	−0.76 (−1.03 to −0.5)
>29 d	163/36,288	115/60,176		1.31 (1.11–1.52)	0.57 (0.46–0.67)	0.43 (0.32–0.53)	56.8 (47.1–67.7)	−0.75 (−0.98 to −0.53)
Indirect effects in the unvaccinated								
Full period	5/403	9/862		1.32 (0.12–2.55)	1.89 (0.3–3.34)	NE	NE	NE
0–14 d	4/403	4/862		6.39 (0.64–12.47)	3.39 (0.63–6.05)	NE	NE	NE
15–21 d	1/394	2/842		0.76 (−0.63 to 2.08)	2.95 (0.97–5.19)	NE	NE	NE
22–28 d	0/386	1/827		0.16 (−0.4 to 0.6)	2.21 (0.72–3.95)	NE	NE	NE
>29 d	0/381	2/778		0.01 (−0.04 to 0.04)	1.2 (−0.97 to 3.12)	NE	NE	NE

*Time since first vaccinated was a proxy of number of vaccine doses and days since last dose. NE, not estimated because of insufficient number of events; RD, risk difference; RR, risk ratio; VE, vaccine effectiveness. †Per 10,000 population per day. ‡Full vaccination.

### Bias-Indicator Analysis

In the bias-indicator analysis, crude risk for infection was much higher for the group in the study period (Appendix Figure 3); estimated crude RR was 1.71 (95% CI 1.62–1.81). This bias was mitigated but not eliminated after adjusting; RR of 1.36 (95% CI 1.27–1.46) showed a higher baseline risk in the study period compared with the reference period.

## Discussion

This study on elderly residents of LTCFs confirms the high benefit of vaccination in this population, reducing the risk for infection by up to 81.8% and avoiding up to 11.6 cases/10,000 population/day in persons with no previous SARS-CoV-2 infection. The risk reduction was through direct protection of vaccinated persons but also through indirect protection of nonvaccinated persons. Those with previous infection also benefited from vaccination, despite an already lower baseline risk in this group.

Immunosenescence and factors related to chronic conditions, together with malnutrition, are known to impair the immunity required for an effective vaccine response (*11*), and lower neutralizing antibody response to Pfizer/BioNTech vaccine in persons >65 years of age has been reported (*12*,*13*). However, our estimates were similar to those of observational studies in younger adult populations. A cohort study of healthcare workers in the United Kingdom found a VE of 70% 21 days after the first dose and 85% 7 days after the second dose of the Pfizer/BioNTech vaccine (*14*). A slightly higher estimate of 94.1% was given in a study with data from Israel (*15*).

Other observational studies have explored VE in older age groups and have found a rate comparable to that seen in younger populations. In a registries-based study from Israel, in persons >70 years of age, VE was 44% at 14–20 days after vaccination, 64% at 21–27 days after vaccination, and 98% at >27 days after the second vaccine dose, rates that were similar to those for younger age groups (*16*). Bernal et al. reported vaccine effects started 10–13 days after vaccination with Pfizer/BioNTech and, in persons >80 years of age, reached 70% >29 days after vaccination and 89% 14 days after the second dose (*17*).

Some studies have focused on LTCFs. A study of COVID-19 outbreaks in skilled nursing facilities in Connecticut, USA, found 63% protection after partial vaccination (14–28 days after the first dose), which is close to our estimates (*7*). A recently released report from the VIVALDI study in the United Kingdom found no protection conferred by the Pfizer/BioNTech vaccine in the first 28 days after the first dose among residents of LTCFs (*8*). Nevertheless, VE during days 29–47 was between 56% and 62%, similar to the range of effect in our study for the period 22–28 days (61.9%). In contrast with these studies, the study from Denmark of LTCFs (I.R. Moustsen-Helms et al., unpub. data) found no protective effect of a first vaccine dose, a 52% reduction of risk in days 0–7 after the second dose, and a 64% reduction after day 7, with a strong confounding effect by calendar time, although no details are provided on the methods for adjustment. An approximation of VE using the screening method in the same population of our study (*18*) also resulted in a reduced VE of 71%, although CIs were wide and compatible with our estimation.

A time-series analysis of surveillance data from Spain comparing SARS-CoV-2 incidence in persons >65 years of age living in LTCFs versus those not living in LTCFs (in whom vaccination did not begin until early February) (*19*) found an 85% (95% CI 81%–88%) reduction in incidence in residents of LTCFs after January 17, which provides further validation of the effect of vaccination in LTCFs. Of note, our work included both symptomatic and asymptomatic infections; risk was probably reduced for both, although to an unknown degree. In national COVID-19 surveillance, 39% of all notified infections since May 10, 2020, in persons >65 years of age were asymptomatic.

A considerable 22% of all participants in our study had a previous documented SARS-CoV-2 infection, although a high number of undocumented infections are possible, especially during the first epidemic wave in March–April 2020. Several studies have documented a high immune response to a first COVID-19 vaccine dose in persons with previous infection (*20*; S. Saadat et al., unpub. data, https://www.medrxiv.org/content/10.1101/2021.01.30.21250843v5; C. Camara et al., unpub. data, https://www.biorxiv.org/content/10.1101/2021.03.22.436441v1). The results of our study add to the literature on this subject by demonstrating that, even though the effect was greater in persons naive to SARS-CoV-2, those with previous infection also benefited from a risk reduction of 57%, although it translated to <1 infection averted/10,000 population/day.

Results from the indirect protection analysis in nonvaccinated persons support the hypothesis that vaccination might reduce transmissibility of SARS-CoV-2 and result in herd immunity. Previous studies have shown decreased viral load in vaccinated persons, including those in LTCFs (*7*,*21*). A study from Scotland found a 30% lower risk for SARS-CoV-2 in household members of vaccinated healthcare workers, although the reduction in SARS-CoV-2 transmission from vaccinated persons could be double that estimate, since household members could also have been infected in the community (A.S. Shah et al., unpub. data, https://www.medrxiv.org/content/10.1101/2021.03.11.21253275v1). A recent ecologic study from Israel has shown that increasing vaccine coverage provides cross-protection to unvaccinated persons in the community (*22*). In our study, nonvaccinated persons living in facilities where most residents and staff had been vaccinated showed a risk reduction similar to persons who were actually vaccinated. However, the magnitude of protection might be overestimated, because nonvaccinated persons could be more likely to have had previous infection, even if not documented. Also, indirect protection was measured in a context of very high vaccine coverage, which is difficult to attain in a noninstitutional setting; therefore, our results might not apply to the community setting.

Some limitations to our study could relate to the before-after comparison. Although we tried to minimize it, the bias-indicator cohort showed residual confounding because of higher incidence during the study period, which coincided with the third epidemic wave. The high incidence could also be related to relaxed isolation in LTCFs during the Christmas season, when numbers of days out and visits were higher. Of note, SARS-CoV-2 testing policy did not change during the study period. This residual bias would be in the direction of underestimation of the protection of the vaccine. Another limitation is that, because the selection of participants was performed through the vaccination registry, we were able to include only persons who survived until the vaccination campaign. We observed a high incidence during the second epidemic wave (9.6% of study participants were infected between the beginning of the reference period and the beginning of the study period). Therefore, the group with previous infection in the study period had more recent infections compared with the group in the reference period; if this factor conferred greater protection, it could overestimate VE in this group. On the other hand, prolonged viral shedding (beyond 60 days) could be mistaken for a new infection and, if this factor was more frequent because of recent infections in the study period, it could decrease VE. However, discarding tests within 90 days (instead of 60) of a previous positive test did not substantially change results (analysis not shown). Finally, full vaccination was accounted for by a proxy of >29 days after the first dose. This assumption is reasonable in our study because uptake of a second dose was very high, and the number of days between doses was 21 for most persons.

A strength of our study was that it included virtually all residents of LTCFs in Spain. The number of included persons was slightly higher than the number of residents in the official LTCF census (299,209 vs. 281,428), which is expected because the census does not include a small number of LTCFs (e.g., those managed by the church).

In conclusion, our results confirm the effectiveness of vaccination in LTCF residents. Our findings endorse the policy of universal vaccination in this setting, including in persons with previous infection, and suggest that nonvaccinated persons benefit from indirect protection. Questions remain regarding the effects of age and previous infection on the duration of protection afforded by vaccination.

Appendix Additional information about direct and indirect effectiveness of mRNA vaccination against severe acute respiratory syndrome coronavirus 2 in long-term care facilities, Spain
