# Supplementary material for: Direct and Indirect Effectiveness of mRNA Vaccination against Severe Acute Respiratory Syndrome Coronavirus 2 in Long-Term Care Facilities, Spain
Source: Emerg Infect Dis. 2021 Oct;27(10):2595–603. doi: 10.3201/eid2710.211184 (PMC8462307; doi:10.3201/eid2710.211184)
Supplement: Appendix — Additional information about direct and indirect effectiveness of mRNA vaccination against severe acute respiratory syndrome coronavirus 2 in long-term care facilities, Spain [file 21-1184-Techapp-s1.pdf]

Article DOI: https://doi.org/10.3201/eid2710.211184

Direct and Indirect Effectiveness of mRNA Vaccination against Severe Acute Respiratory Syndrome Coronavirus 2 in Long-Term Care Facilities, Spain

Appendix

Appendix Table 1. Crude estimations of risk ratio and risk difference in residents of long-term care facilities according to evidence of previous severe acute respiratory syndrome coronavirus 2 infection and time since first vaccinated (as a proxy of number of vaccine doses and days since last dose), Spain, December 27, 2020–March 10, 2021*

Time since vaccination	RR		RD (per 10,000 persons/day)	
	No previous infection	Previous infection	No previous infection	Previous infection
Effects in the vaccinated				
Full period	0.53 (0.95–0.52)	0.66 (0.57–0.74)	−5.48 (−5.68 to −5.28)	−0.59 (−0.8 to −0.39)
0–14 d	1.06 (0.95–1.04)	1.06 (0.87–1.25)	1.13 (0.66–1.58)	0.15 (−0.28 to 0.59)
15–21 d	0.65 (0.95–0.64)	0.82 (0.7–0.93)	−5.22 (−5.49 to −4.96)	−0.39 (−0.67 to −0.11)
22–28 d	0.48 (0.95–0.47)	0.69 (0.59–0.78)	−7.03 (−7.26 to −6.8)	−0.61 (−0.85 to −0.38)
≥29 d	0.2 (0.95–0.19)	0.4 (0.31–0.49)	−7.33 (−7.53 to −7.12)	−0.85 (−1.09 to −0.63)
Indirect effects in the unvaccinated				
Full period	0.42 (0.32–0.52)	NE	−10.03 (−12.65 to −7.4)	NE
0–14 d	0.92 (0.68–1.15)	NE	−1.62 (−6.52 to 3.37)	NE
15–21 d	0.59 (0.45–0.72)	NE	−8.05 (−11.19 to −4.83)	NE
22–28 d	0.44 (0.33–0.54)	NE	−10.46 (−13.23 to −7.67)	NE
≥29 d	0.18 (0.11–0.25)	NE	−12.89 (−15.88 to −9.91)	NE

*NE, not estimated because of insufficient number of events; RD, risk difference; RR, risk ratio Data used for these calculations are similar to data in Tables 1 and 2 in the main article, but unadjusted estimates are provided here.

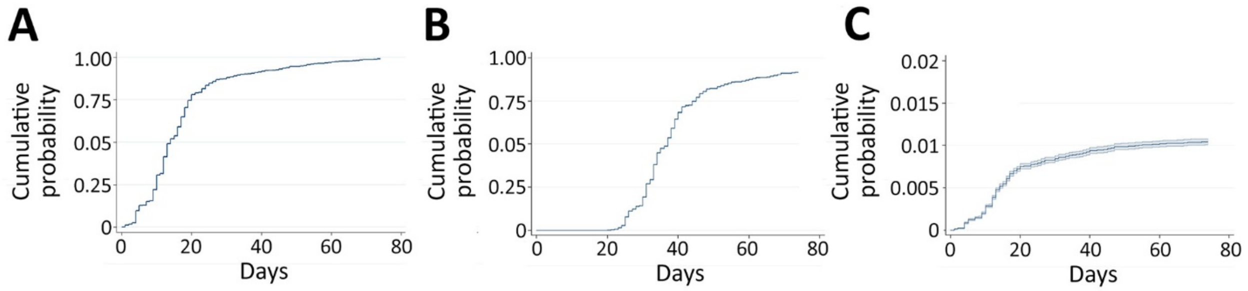

Appendix Figure 1. Vaccination uptake in residents of long-term care facilities (N = 299,209) included in study of effectiveness of mRNA vaccine against severe acute respiratory syndrome coronavirus 2, Spain, December 27, 2020–March 10, 2021. A) Time to first vaccine dose, B) time to second vaccine dose, C) time to first offer of vaccine for nonvaccinated persons.

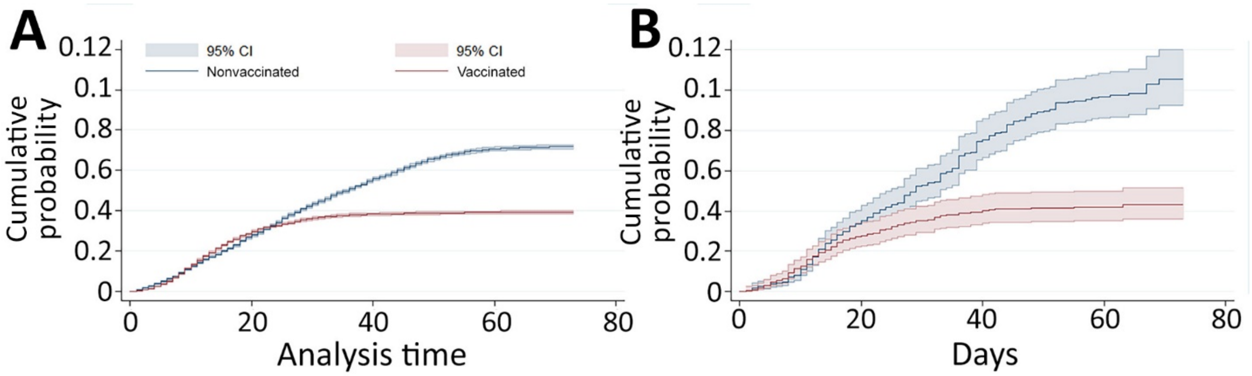

Appendix Figure 2. Unadjusted Kaplan-Meier estimates of the cumulative risk for a positive diagnosis of severe acute respiratory syndrome coronavirus 2 (SARS-CoV-2) in elderly residents of long-term care facilities, Spain. A) Risk for SARS-CoV-2 infection in vaccinated (i.e., in the study period) and nonvaccinated (i.e., in the reference period) persons, B) risk for SARS-CoV-2 infection in indirectly protected (i.e., nonvaccinated in the study period) and nonvaccinated (i.e. nonvaccinated in the reference period).

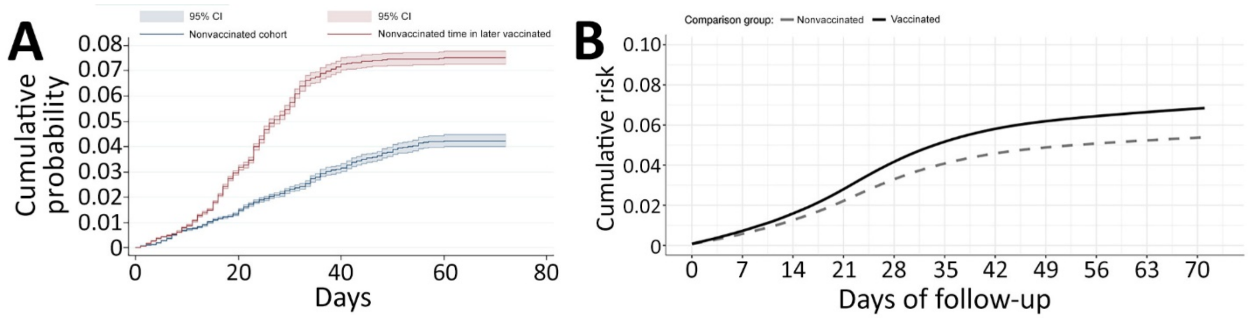

Appendix Figure 3. Unadjusted and adjusted estimates of cumulative risk for severe acute respiratory syndrome coronavirus 2 in residents of long-term care facilities, Spain. A) Unadjusted Kaplan-Meier estimates of cumulative risk in the bias indicator cohort and B) adjusted estimates of cumulative risk.
